# Biportal endoscopic lumbosacral foraminotomy in dogs: technical feasibility and anatomical assessment in cadavers

**DOI:** 10.3389/fvets.2025.1637089

**Published:** 2025-09-05

**Authors:** Jiyoon Kim, Haebeom Lee, Jaemin Jeong, YoungJin Jeon

**Affiliations:** Department of Veterinary Surgery, College of Veterinary Medicine, Chungnam National University, Daejeon, Republic of Korea

**Keywords:** biportal endoscopic spine surgery, lumbosacral foraminal stenosis, lumbosacral foraminotomy, minimally invasive spine surgery, degenerative lumbosacral disease

## Abstract

**Introduction:**

Lumbosacral foraminal stenosis (LSFS) in dogs, a subtype of degenerative lumbosacral disease, leads to nerve root compression and clinical signs such as pain or pelvic limb dysfunction. Traditional decompression techniques—such as dorsal laminectomy or lateral foraminotomy—have limitations, including restricted access, tissue disruption, and prolonged recovery. To address these challenges, this study evaluated the technical feasibility and safety of biportal endoscopic lumbosacral foraminotomy (BELF), a minimally invasive technique adapted from human unilateral biportal endoscopy (UBE), for decompressing the lumbosacral foramina in canine cadavers.

**Methods:**

BELF was performed bilaterally on nine canine cadavers (18 foramina) after initial refinement in a pilot study using three cadavers. Key outcome measures included computed tomography (CT)-based measurements of foraminal area at the entry, middle, and exit zones pre-and postoperatively, intraoperative endoscopic video evaluation of anatomical visualization and surgical performance, and a feasibility scoring system (ratings: Excellent, Good, Fair, Poor).

**Results:**

BELF was successfully completed in all specimens without causing damage to normal anatomical structures or requiring conversion to open surgery. The mean operative time was 42.17 ± 13.27 min. Postoperative CT showed significant foraminal enlargement at all level; entry (+53.8%), middle (+81.6%), and exit (+119.8%) compared to preoperative measurements (all *p* < 0.001). Critical anatomical structures were preserved with clear endoscopic visualization, and all procedures were rated as Excellent or Good on the feasibility scale.

**Conclusion:**

BELF enabled effective and minimally invasive decompression of the lumbosacral foramina with no observed damage to critical anatomical structures. These results support the feasibility of BELF and its potential clinical utility in managing LSFS in dogs.

## 1 Introduction

Lumbosacral foraminal stenosis (LSFS), a subtype of degenerative lumbosacral stenosis, is characterized by progressive narrowing of the intervertebral foramina, leading to compression of the exiting nerve root. This compression often results in clinical signs such as lower back pain and, in severe cases, neurological deficits such as root signatures and/or dragging of the affected pelvic limb (1, 2). Management strategies include conservative medical therapy and surgical intervention, with surgical decompression indicated when conservative treatments fail to provide sufficient relief (3, 4). Surgery is often the preferred option for working or performance dogs that need to return to activity quickly (1, 4, 5).

Surgical treatment for LSFS primarily involves bony decompression through foraminotomy and/or dorsal laminectomy (5, 6). Several studies have described dorsal or lateral approaches for decompressing the L7 nerve root (7, 8). However, dorsal approaches have inherent limitations, including restricted surgical fields and risks of postoperative hypermobility, instability, and contralateral facet fractures (9, 10). In addition, foraminotomy through the lateral approach requires extensive dissection of the paraspinal muscles and associated ligaments, including the multifidus, sacrocaudalis dorsalis, quadratus lumborum, and longissimus muscles (10). Such extensive soft tissue manipulation contributes to increased postoperative pain, inflammation, and prolonged recovery (11). Additionally, the anatomical constraints of the lumbosacral (LS) region, particularly the iliac wing, challenge achieving adequate exposure via a lateral approach (11–13). To improve surgical access, modifications such as cranial iliac wing osteotomy and transiliac approach have been proposed (13). While these techniques enhance visualization, they also increase surgical complexity, operating time, and the risks of additional pain, bleeding, and the necessity for bone healing and stabilization (12, 13). Given these challenges, there is an increasing demand for advanced surgical techniques that effectively achieve decompression while minimizing the risk of complications.

In response to these limitations, minimally invasive techniques have gained interest in veterinary spinal surgery. Various endoscopic approaches for spine surgery have been introduced to reduce soft tissue disruption and improve visualization (14, 15). However, reports specifically applying endoscopic techniques to LS foraminotomy remain limited. To date, a few studies have described the use of endoscopic-assisted LS foraminotomy in dogs, demonstrating the potential value of endoscopic visualization as an adjunct to conventional open techniques (6, 12). Among these methods, unilateral biportal endoscopy (UBE) spine surgery has emerged as a promising alternative to traditional open and other minimally invasive techniques by allowing a high degree of visualization while reducing surgical trauma. In human spinal surgery, biportal endoscopic techniques have been successfully utilized for lumbar decompression, including foraminal stenosis, and have demonstrated favorable clinical outcomes (16–19). Unlike uniportal endoscopic techniques, UBE provides a broad surgical field and effective decompression by utilizing two separate portals. One portal is dedicated to endoscopic visualization, while the other is used for instrument manipulation. This configuration functions similarly to arthroscopy and allows for superior fluid outflow through the working portal. This technique supports the use of conventional arthroscopic and spinal surgical instruments, making it easier for surgeons with experience in open procedures to adapt (20–22).

This study aimed to describe surgical techniques and to evaluate the feasibility and safety of biportal endoscopic lumbosacral foraminotomy (BELF) for the treatment of canine LSFS using cadavers. We hypothesized that BELF would provide improved visualization of key anatomical structures while minimizing iatrogenic injury to the facet joints, neural elements, and surrounding soft tissues, and the technique would result in adequate enlargement of the lumbosacral foramen.

## 2 Materials and methods

### 2.1 Sample population and study design

Twelve canine cadavers were used in this study. The first three cadavers were used in a pilot study to optimize portal placement for the BELF procedure and were not included in the final data analysis. All cadavers were obtained from dogs that had been enrolled in unrelated terminal studies approved by the Institutional Animal Care and Use Committee of Chungnam National University (Approval Nos: 202304A-CNU-011 and 202404A-CNU-066). Radiography and computed tomography (CT) were performed to rule out lumbosacral joint disease and plan the portal placement. Both sides of the lumbosacral foramen were used for the study, resulting in a total of 18 experimental cases. The cadavers were stored at −20 °C and then thawed at room temperature (approximately 22 °C) a day before the experiment. All procedures were performed by a single right-handed orthopedic surgeon (HL), with over 15 years of experience in arthroscopy. Body weight and body condition score of the cadavers were measured prior to the procedure.

### 2.2 Computed tomography evaluation

CT scans were performed with a slice thickness of 0.5 mm using an 80-row MDCT (Aquillion Prime SP, TSX-303B; Canon Medical Systems, Japan). The cadavers were positioned in ventral recumbency, with their limbs naturally arranged in a frog-legged posture (23). Following the method reported in a previous study, the parasagittal foramen area (PFA) at the entry, middle, and exit zones was systematically measured using CT images and a specialized software program (RadiAnt DICOM Viewer, Medixant, Poznań, Poland) (6). These measurements served as the baseline for comparison of foraminal enlargement after BELF.

### 2.3 Surgical instruments

**Figure 1:**
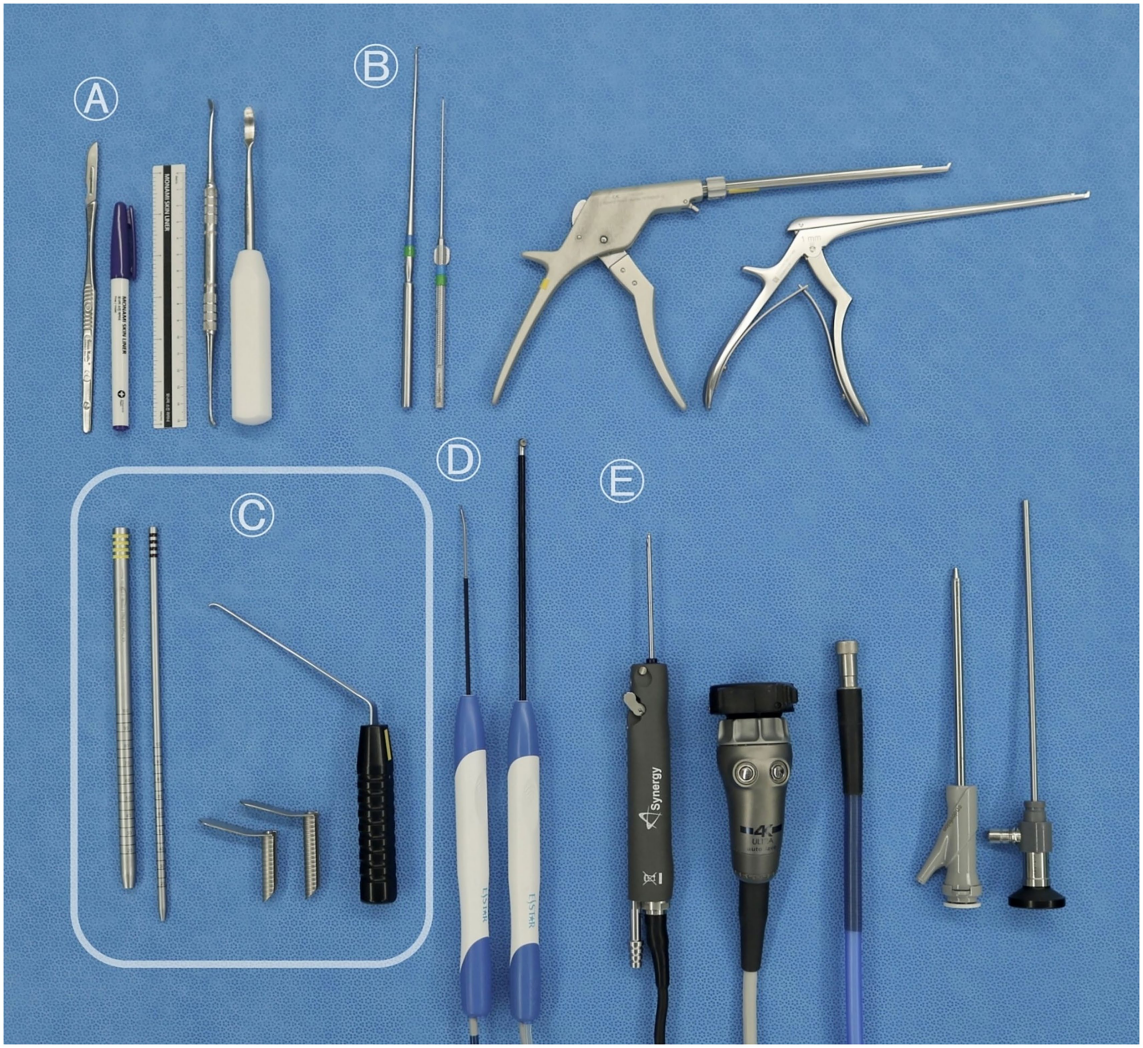
Surgical instruments used for the biportal endoscopic lumbosacral foraminotomy procedure. **(A)** Common orthopedic instruments: scalpel, No. 10 blade, surgical pen, ruler, and periosteal elevator. **(B)** Conventional neurosurgical instruments: nerve hook probes, 2-mm rotating Kerrison rongeur, and 1-mm Kerrison rongeur. **(C)** Instruments specifically required for unilateral biportal endoscopy: serial portal dilators for creating the working channel, semi-tubular retractors to maintain fluid outflow, and a root retractor. **(D)** Optional arthroscopic tools occasionally used for soft tissue removal and coagulation: bipolar radiofrequency probes. **(E)** Standard arthroscopic instruments: a 3.0-mm hooded round burr, a camera and light cable, and a 0° 4.0-mm endoscope.

The following surgical instruments were used in this study: a vacuum positioning bag; 18-gage needles; a No. 10 blade; a surgical pen with a flexible plastic ruler; periosteal elevators; nerve hook probes; a 2 mm rotational Kerrison rongeur (Solendos, Seoul, Republic of Korea); a 1 mm Kerrison rongeur (Integra Life Sciences, Princeton, NJ); serial portal dilators (Solendos); a semi-tubular retractor (Solendos); a root retractor (Endovision, Daegu, Republic of Korea); a radiofrequency generator (RF; FRG-100B, Biounit, Hanam, Republic of Korea); a 3 mm hooded round burr (AR-9300RBT, Arthrex, Naples, FL); a camera and light cable (Synergy, Arthrex); and a 4.0 mm, 0° endoscope (SPINUSS SCOPE, Endovision) (Figure 1).

### 2.4 Surgical procedure

#### 2.4.1 Cadaver positioning

The cadaver was positioned in ventral recumbency with the hind limbs slightly flexed. An iodine-impregnated incise drape (Ioban, 3 M Healthcare, Saint Paul, MN) was applied to the skin surface. A disposable surgical drape specifically designed for unilateral biportal endoscopy (SJ-GLID 901, Sejong Healthcare, Paju, Republic of Korea) was then placed over the operative field.

#### 2.4.2 Localization and portal creation

**Figure 2:**
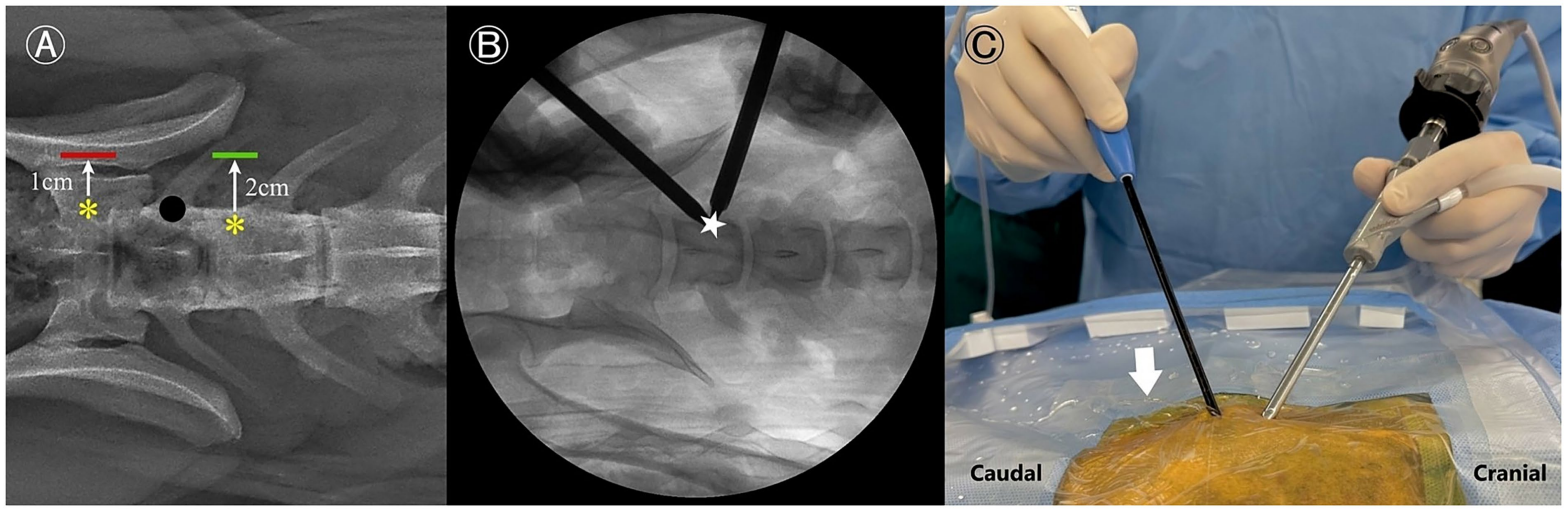
Portal creation process for a left-sided approach. **(A)** Radiographic dorsoventral view illustrating the reference points for skin incision planning. The working portal is indicated by a red line, and the viewing portal is indicated by a green line. Anatomical reference points are marked with yellow asterisks, and the docking point is represented by a black dot. **(B)** Fluoroscopic image showing the recommended docking point (star), positioned just caudal to the L7 cranial articular process or on the dorsal aspect of the L7 transverse process. **(C)** Intraoperative macroscopic view demonstrating fluid outflow (arrow) through the instrument portal.

Portal positions were determined by adapting human spinal anatomical landmarks to canine anatomy (19). A paraspinal approach was employed, with fluoroscopy guiding landmark identification. The cranial end of the cranial articular process (AP) and the caudal end of the caudal AP of L7 were identified and marked using 18-gage needles under fluoroscopic guidance, which served as the reference points. The cranial and caudal portals, 7 mm and 10 mm in length, were established 2 cm and 1 cm lateral to the reference points, respectively. Two incisions were made using a blade to create these portals. The cranial portal was designated as the viewing portal, and the caudal portal served as the working portal. The docking point was located at the midpoint of the transverse process-vertebral body junction of L7 to avoid injury to the extraforaminal nerve root (Figure 2A).

**Figure 3:**
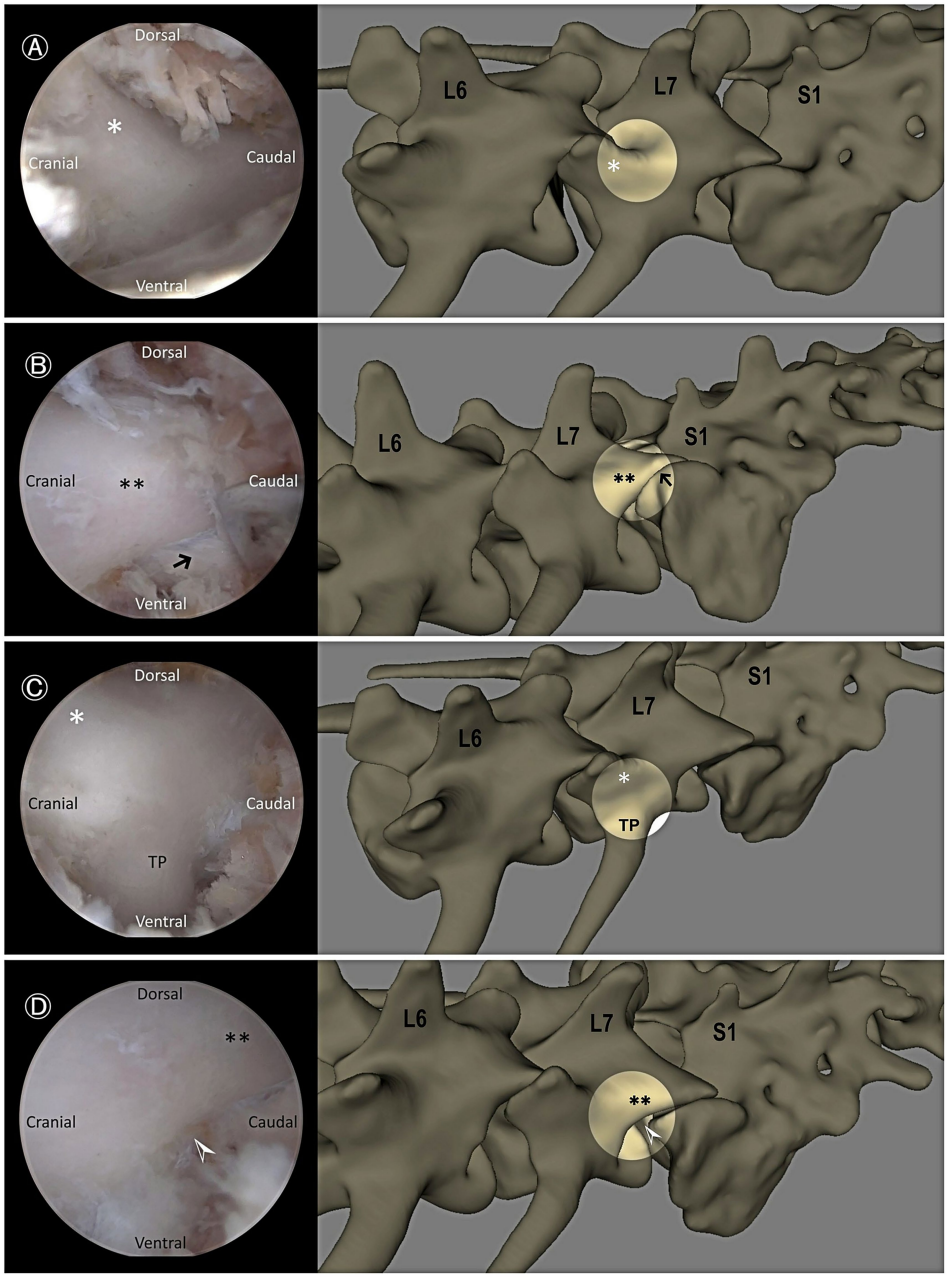
Endoscopic views demonstrating key anatomical landmarks. **(A)** Cranial articular process of L7 (asterisk). **(B)** Caudal articular process of L7 (double asterisks) and the facet joint between L7 and the sacrum (black arrow). **(C)** Cranial articular process of L7 (asterisk) and dorsocaudal aspect of the transverse process (TP) of L7. **(D)** Caudal articular process of L7 (double asterisks) and the lumbosacral foramen (white arrowhead).

The arthroscopic sheath with obturator was inserted through the viewing portal, and a periosteal elevator was introduced through the working portal. The epaxial muscles were gently elevated from the pedicle using blunt dissection. Once muscle elevation was achieved, a serial dilation sleeve was then advanced through the working portal, and its position was confirmed using fluoroscopy (Figure 2B). If necessary, a semi-tubular retractor was positioned over the working sleeve to stabilize the portal and optimize continuous fluid egress. The obturator was then withdrawn from the endoscopic sheath, and a camera with an integrated light source was inserted into the sheath. For optimal irrigation, water pressure was maintained below 30 mmHg by suspending a 3 L bag of normal saline 40 cm above the surgical site (20, 24). Passive fluid outflow is maintained by the pressure gradient created by gravity-assisted irrigation (Figure 2C). The residual soft tissue was removed as needed using an RF probe. During this step, the cranial and caudal APs of L7, the dorsocaudal origin of the transverse process (TP), and the lumbosacral foramen along the pedicle were identified (Figure 3).

#### 2.4.3 Endoscopic bone work

**Figure 4:**
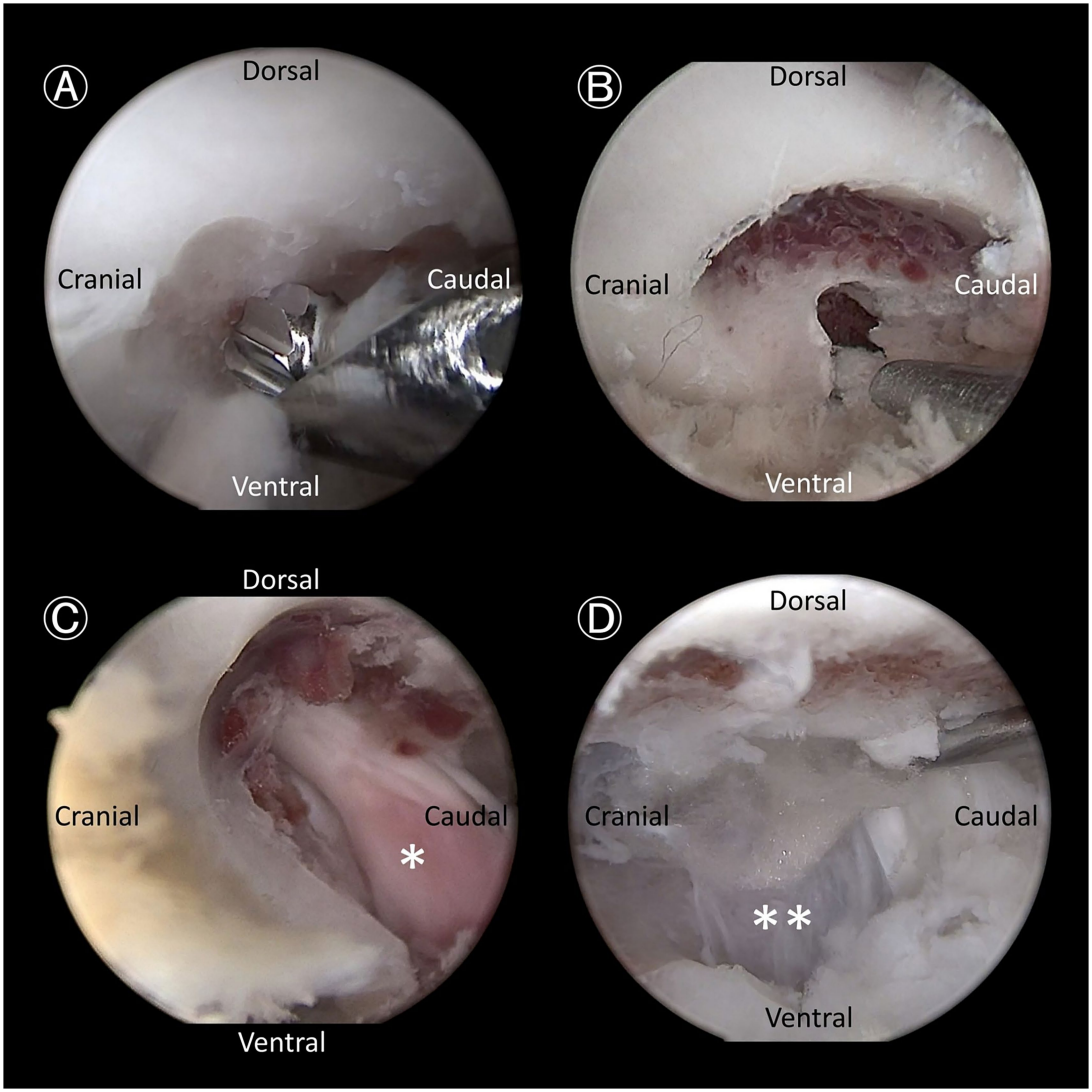
Endoscopic views demonstrating key steps. **(A)** Bone removal using a hooded bur, designed to prevent soft tissue entrapment and nerve root injury, as it removes the outer cortical and cancellous bone. **(B)** Further bone removal with a 1-mm Kerrison rongeur, targeting the cancellous bone and inner cortical bone. **(C)** Expanded intervertebral foramen with exposure of the L7 nerve root ganglion (asterisk). **(D)** Retraction of the L7 nerve root using a probe to expose the annulus fibrosus of the lumbosacral disc (double asterisks).

Based on bone work guidelines from established conventional surgical techniques (10), the height of the drill hole extended from the dorsal aspect of the TP to the base of the cranial AP of L7. The length of the foraminotomy extended from the caudo-dorsal origin of the TP into the exit zone of the intervertebral foramen. First, bone was removed until only the inner cortical layer remained using a 3 mm hooded burr (Figure 4A). The foraminotomy was further extended cranially and dorsally using a burr and/or 1–2 mm Kerrison rongeurs (Figure 4B). After exposing the entry zone of the lumbosacral foramen, a nerve hook probe was used to delineate the extent of the canal and to identify and mobilize the neural tissue (Figure 4C).

### 2.5 Intraoperative assessment

Operative time was recorded from the initial identification of anatomical landmarks to final closure. In the axial plane, the scope angle was defined as the angle between the endoscope and a dorsal reference line representing the direction of the spinous process (SP), which was established using a spinal needle. The instrument angle was similarly defined as the angle between the axis of the surgical instrument and the same SP-based reference line. The triangulation angle was measured as the angle between the endoscope and the surgical instrument, viewed from the lateral side opposite the surgical approach.

Feasibility of the BELF technique was assessed by an independent evaluator (JK) using real-time intraoperative endoscopic video monitoring, based on a predefined scoring system comprising two domains: anatomical visualization and accessibility, and surgical performance.

#### 2.5.1 Visualization and accessibility of anatomical landmarks

Seven anatomical landmarks were evaluated, and each structure was assessed based on whether it was clearly visualized and could be easily accessed using a probe. The assessed landmarks included the following: the base of the cranial AP of L7, the caudal AP of L7, the caudo-dorsal origin of the TP, the LS foramen, the L7 nerve root, the cauda equina, and the dorsal surface of the LS annulus fibrosus (Figure 4D). The total number of landmarks successfully visualized and accessed determined the score in this domain (Table 1). Endoscopic identification and accessibility of all anatomical landmarks are demonstrated in Supplementary Video S1.

**Table 1:** Feasibility evaluation criteria for BELF procedure.

Score	Visualization / Accessibility	Endoscopic disorientation	Soft tissue interference	Tissue discrimination	Surgical time
0	4 or fewer landmarks	≥ 3 times	Severe interference despite radiofrequency (RF) use	No distinction between tissues	> 58 min ^a^
1	5 landmarks	2 times	Persistent but manageable obstruction	Poor distinction; injury occurred	31–58 min
2	6 landmarks	1 time	Minimal interference, resolved with RF	Achievable with caution	≤ 30 min ^b^
3	7 landmarks	None	No interference	Clear distinction	–

a Based on the average operative time reported in a cadaveric study on uniportal endoscopic mini-hemilaminectomy in dogs (14).

b Based on the average operative time reported in a cadaveric study on uniportal endoscopic limited-lumbosacral-dorsal laminectomy study in dogs (15).

#### 2.5.2 Surgical performance

This domain consisted of four subcategories, each scored on a predefined scale: endoscopic disorientation, calculated by summing the number of viewing portal installations and dorsal slippage events during the procedure; soft tissue interference; tissue discrimination; and surgical time, evaluated based on predefined reference thresholds. Each subcategory was rated on a 0–3 scale, except for surgical time, which was scored on a 0–2 scale.

Surgical time was evaluated using a 3-point ordinal scale based on absolute duration and external benchmarks. A score of 0 was assigned to procedures lasting ≥ 58 min, corresponding to the average operative time reported in a cadaveric study on uniportal endoscopic mini-hemilaminectomy in dogs (14). A score of 2 was assigned for procedures ≤ 30 min, reflecting the average procedural time reported in a cadaveric study on uniportal endoscopic limited-lumbosacral-dorsal laminectomy study in dogs (15). Procedures lasting 31–57 min were assigned a score of 1. This classification enabled standardized grading of procedural efficiency, independent of relative rankings within the dataset.

The overall feasibility score was determined by adding the scores from both domains and classified as follows: Excellent (12–14 points), Good (9–11 points), Fair (6–8 points), and Poor (0–5 points).

### 2.6 Post-operative evaluation

The portal incision length was measured postoperatively, along with the lateral distances from the spinal midline for each incision and the inter-incisional distance. A postoperative CT scan was conducted to assess the extent of foraminal enlargement by comparing the foramen area before and after foraminotomy. The scan was performed consistently with the preoperative protocol. The multi-plane foraminal area (PFA) was measured at the entry, middle, and exit zones.

### 2.7 Statistical analysis

Data analysis was performed using SPSS (version 26.0; IBM Corp). The Shapiro–Wilk test was used to assess the normality of continuous variables, and variables following a normal distribution were expressed as mean ± standard deviation. Paired t-tests were used to compare the differences in the degree of foraminal enlargement between preoperative and postoperative measurements at the entrance, middle, and exit zones, and the incision lengths between the working and viewing portals. The Wilcoxon signed-rank test was used to evaluate differences in axial angle between the working and viewing portals, and comparisons between left-and right-sided procedures in terms of viewing portal incision length, working portal incision length, scope installation number, individual parameters of surgical performance assessment, and the total feasibility evaluation score. Robust regression analysis (Huber M-estimator) was used to evaluate the association between surgical time and the number of sequential cases performed (R-4.5.1 for Windows, R Foundation for Statistical Computing, Vienna, Austria). For all statistical analyses, a *p* value of < 0.05 was considered statistically significant.

## 3 Results

### 3.1 Pre-operative measurements

The cadaver specimens, all of which were beagles, had a median body weight of 10.85 kg (range: 9.85–11.25 kg) and a median body condition score of 7 (range: 5–7) on a 9-point scale.

### 3.2 Intraoperative assessment

BELF could be performed in all specimens without procedural failure. The mean operative time was 42.17 ± 13.27 min. Robust regression analysis demonstrated a significant negative association between sequential case number and surgical time (slope = −1.09, *p* = 0.027). The mean axial scope angle was 34.47 ± 9.21°, while the mean axial instrumental angle measured 31.38 ± 7.36°. The mean triangulation angle was 39.02 ± 9.11°. There were no significant differences between the working portal and the viewing portal in terms of axial angle (*p* = 0.248).

Three cases exhibited dorsal mispositioning of the docking site relative to the intended target. This was immediately corrected using fluoroscopic guidance, and no further mispositioning occurred. No damage to adjacent structures, including the APs, TP, or iliac wings, was observed in any case.

#### 3.2.1 Feasibility scores

All anatomical landmarks were successfully visualized before the bone work. Endoscopic disorientation showed a statistically significant difference, with higher scores on the right side compared to the left (*p* = 0.020). Soft tissue interference, tissue discrimination, and surgical time did not show statistically significant differences between sides (*p* = 0.157, *p* = 1.00, and *p* = 1.00, respectively). Total scores did not differ significantly between sides (*p* = 0.257). Among the 18 sides assessed, 7 out of 18 sides were rated as Excellent, and 11 out of 18 sides were rated as Good.

### 3.3 Post-operative measurements

Incision measurements showed a mean viewing portal incision length of 9.33 ± 2.38 mm and a mean working portal incision length of 10.22 ± 3.19 mm (Table 2). The mean distance from the spinal midline to the incisions was 21.22 ± 5.25 mm, while the mean distance between the two incisions was 23.28 ± 3.63 mm (Table 2). There were no significant differences in incision length or inter-portal distance between the left-and right-sided procedures, except for the viewing portal incision length. The viewing portal incision length was significantly longer when the scope was held with the non-dominant hand (median: 10 mm, range: 7–15 mm) compared to the dominant hand (median: 8 mm, range: 6–12 mm; *p* = 0.049), corresponding to left-and right-sided procedures, respectively. The working portal incision length did not differ significantly between dominant (median: 10 mm, range: 6–16 mm) and non-dominant hand use (median: 10 mm, range: 6–15 mm; *p* = 0.943). There were no significant differences between the working portal and the viewing portal in terms of incision length (*p* = 0.287). The number of endoscopic portal installations was significantly higher for the non-dominant hand than for the dominant hand (*p* = 0.02).

**Table 2:** Surgical time and postoperative incision data.

Case no. ^a^	Surgical time (min)	Postoperative incision length (mm)		Inter-incisional distance (mm)
		Viewing portal	Working portal	
1	80	8	9	28
2	39	12	8	22
3	45	12	16	20
4	56	7	6	22
5	30	9	8	24
6	33	8	14	27
7	43	15	10	19
8	58	8	15	25
9	32	10	15	22
10	50	6	10	20
11	39	7	6	30
12	43	11	10	25
13	37	9	7	25
14	50	10	11	21
15	36	6	8	27
16	35	12	12	23
17	30	9	7	24
18	23	9	12	15
Mean ± SD	42.17 ± 13.27	9.33 ± 2.38	10.22 ± 3.19	23.28 ± 3.63

a Case numbers correspond to the chronological order of procedures, from the first to the last surgery.

**Figure 5:**
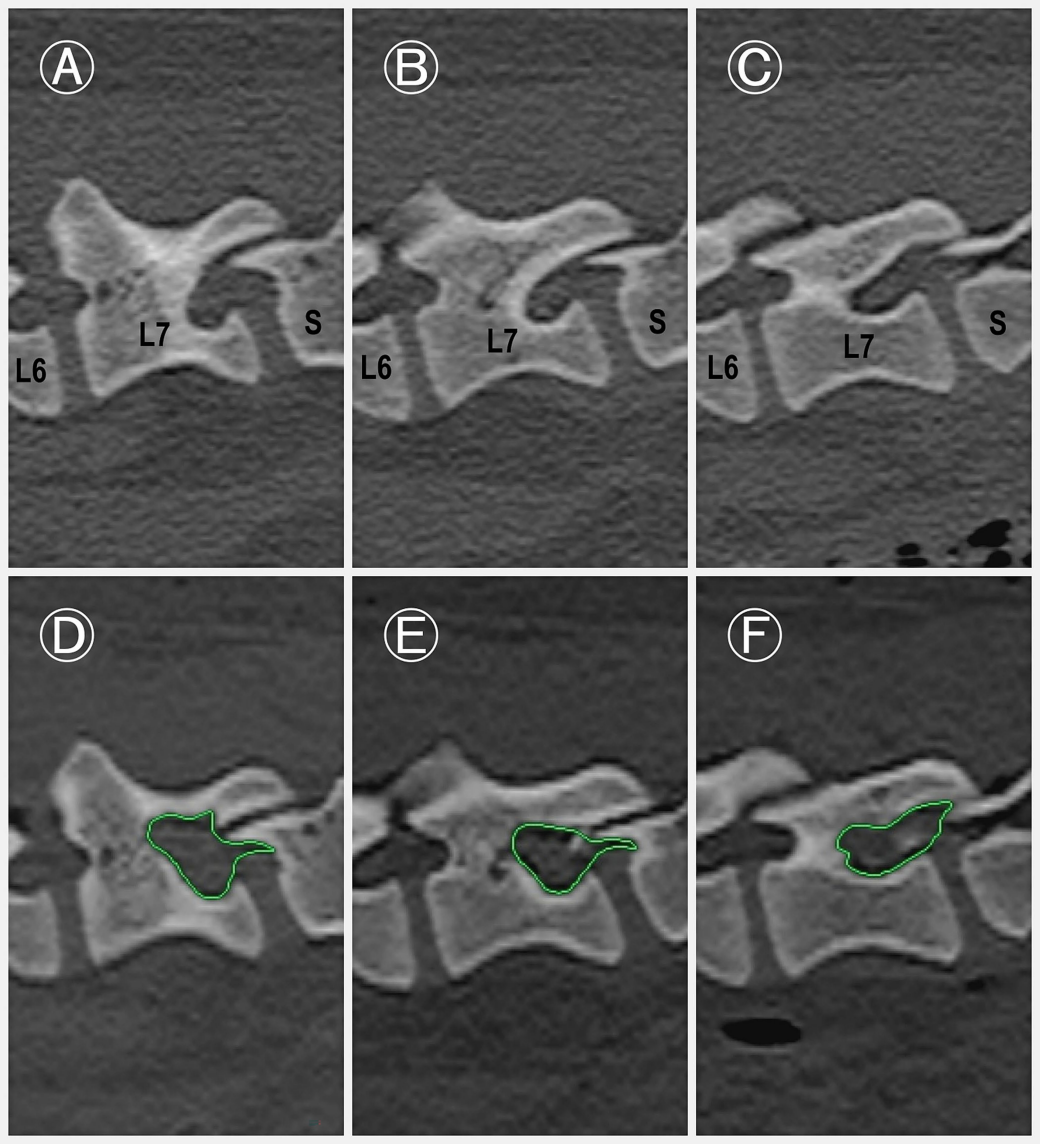
Pre-and postoperative para-sagittal CT images of the L7–S1 intervertebral foramen. **(A–C)** Preoperative images of the L7–S1 intervertebral foramen at three anatomical levels: exit zone **(A)**, middle zone **(B)**, and entrance zone **(C)**. **(D–F)** Corresponding postoperative images obtained immediately after the procedure, demonstrating notable enlargement of the foraminal space. The increase in area (green highlighted area) is particularly evident in the middle **(E)** and exit **(D)** zones.

Foraminal dimensions increased by 153.80 ± 15.92% in the entrance zone, 181.61 ± 32.45% in the middle zone, and 219.80 ± 38.00% in the exit zone (Figure 5; Supplementary Table S1). The foraminal size significantly increased in the entrance, middle, and exit zones compared to the preoperative condition (*p* < 0.001 for all zones).

## 4 Discussion

In this study, we developed and described a novel technique, BELF, as a minimally invasive alternative to the conventional LS foraminotomy by adapting a human surgical procedure. Compared to traditional open surgery, endoscopic techniques offer several advantages, including magnified visualization, improved anatomical localization, and minimized soft tissue disruption. These features were central to assessment of the BELF approach in this study. To validate the feasibility and safety of this approach, we conducted cadaveric evaluations. The results confirmed that the BELF technique could be performed successfully without causing additional damage to the facet joint or ilium. Moreover, the procedure allowed for adequate enlargement of the lumbosacral foramen, clear visualization, and efficient instrument handling. These findings support the potential of the BELF technique as a minimally invasive option for LS foraminotomy in veterinary practice.

In human literature, a clear understanding of the relevant anatomical landmarks and procedural guidelines has been considered extremely important, and extensive research has been conducted on the topic (18, 19, 21, 25–27). This emphasis arises from the fact that failure to follow these guidelines may result in complications such as prolonged surgical times, infections, postoperative hematomas, and dural tears (21). Our study aimed to clarify these principles by providing a detailed description of the BELF, based on current literature (19). However, direct application of human surgical methods to canine patients is impractical due to significant anatomical differences. Notably, the canine iliac wing differs from that of humans; dogs possess a wider pelvis with more laterally oriented iliac crests, which affects musculature and overall biomechanics (28). In human lumbosacral surgery, the paraspinal skin approach typically utilizes the vertebral isthmus as a docking point. However, this structure is not well-developed in dogs (21). Additionally, canine lumbar TP projects more laterally, is significantly longer and narrower than in humans, and does not connect directly to the dorsal arch (29). Given these anatomical distinctions, we modified the surgical approach by designating the caudal border of the cranial AP as the docking point. This modification allowed for reliable anatomical reference points while maintaining procedural feasibility. To refine portal creation, we followed existing guidelines to determine appropriate incision lengths. Incisions were made at 7 mm for the endoscopic portal and at 10 mm for the working portal to ensure proper instrument insertion and smooth saline flow (21).

Following the application of the BELF, the procedure resulted in significant foraminal enlargement (*p* < 0.001) with minimal anatomical disruption to the spine. The average percentage increase in foraminal dimensions was measured across three zones: the entrance zone (+53.80 ± 15.92%), the middle zone (+81.61 ± 32.45%), and the exit zone (+119.80 ± 38.00%). To date, no comparative data are available in in veterinary study. However, a human clinical study involving stenotic foramina reported an average 45.5% increase in foraminal area following lumbosacral foraminotomy, with acceptable *in vivo* clinical outcomes (30). Notably, the results indicate that the procedure was particularly effective in expanding the middle and exit zones. Clinically, this is relevant because a previous study found that foraminal stenosis occurred in the middle zone in 20% of cases, while 65% showed stenosis in both the middle and exit zones (10). Given these distributions, achieving adequate enlargement in middle and exit zones with the BELF technique can lead to clinically meaningful surgical outcomes in most cases.

During the procedure, technical observations revealed specific challenges related to portal positioning and hand dominance. Dorsal slippage resulting in docking point misorientation occurred in three cases (3/18), all of which involved scope manipulation using the non-dominant hand. No significant differences were found between the cranial and caudal portals in terms of axial angle, incision length, or the number of portal installations. However, when the scope was held with the non-dominant hand, the viewing portal tended to have a longer incision length and required more frequent portal repositioning, indicating increased procedural complexity under these conditions. In addition, surgical times showed a decreasing trend as the procedure was repeated. The first case required the longest operative time, followed by a gradual reduction across subsequent cases. Robust regression analysis demonstrated a significant reduction of approximately 1.09 min per case (*p* = 0.027), confirming a learning curve effect. These results suggest that operative efficiency improved with procedural repetition.

Several limitations of this study should be acknowledged. First, the procedures were performed on cadaveric specimens with anatomically normal L7 foramina, rather than on clinically affected cases with lumbosacral foraminal stenosis. Consequently, the technical difficulty and surgical challenges may have been underestimated compared to clinical scenarios. While the BELF technique appeared to minimize soft tissue disruption, this conclusion was based solely on cadaveric observations. The absence of post-procedural MRI evaluation leaves uncertainty regarding the true extent of soft tissue injury, including potential muscle edema, fibrotic changes, or fluid accumulation. Moreover, cadaveric models do not reproduce critical intraoperative variables such as hemorrhage, neural irritation, or physiological responses, which may affect the complexity and safety of the procedure in clinical settings. Therefore, further *in vivo* studies are warranted to validate the safety, hemostatic control, and long-term outcomes of the BELF technique under realistic surgical conditions. Second, all specimens were from Beagle dogs, a breed not commonly predisposed to LSFS. Variations in pelvic morphology among breeds may affect portal mobility and procedural complexity, limiting the generalizability of the findings. Third, all procedures were performed by a single surgeon, which may have introduced operator-specific factors such as dominant-hand handling preferences or technique consistency. Further studies involving larger sample sizes and multiple surgeons are warranted to validate the reproducibility and clinical applicability of the BELF technique.

Despite these limitations, this study confirms the feasibility of the BELF technique in canine cadavers, demonstrating adequate enlargement, clear endoscopic visualization, and preservation of anatomical structures. Future *in vivo* studies involving clinical cases and multiple operators are needed to confirm these findings and to assess long-term safety and clinical outcomes.

## Data Availability

The raw data supporting the conclusions of this article will be made available by the authors, without undue reservation.

## Supplementary material

SUPPLEMENTARY VIDEO S1 Representative intraoperative endoscopic video of the biportal endoscopic lumbosacral foraminotomy (BELF) procedure.
