# Supplementary material for: Biportal endoscopic lumbosacral foraminotomy in dogs: technical feasibility and anatomical assessment in cadavers
Source: Front Vet Sci. 2025 Sep 5;12:1637089. doi: 10.3389/fvets.2025.1637089 (PMC12446017; doi:10.3389/fvets.2025.1637089)
Supplement: Supplementary file 2 [file Table_1.DOCX]

|  | **Pre-operative measurement (cm^2^)** | | | **Post-operative measurement (cm^2^)** | | | **Enlargement** ^a^ **(%)** | | | |
| --- | --- | --- | --- | --- | --- | --- | --- | --- | --- | --- |
| **No.** | **Entrance** | **Middle** | **Exit** | **Entrance** | **Middle** | **Exit** | **Entrance** | **Middle** | **Exit** |  |
| **1** | 0.2637 | 0.2539 | 0.2319 | 0.4309 | 0.4824 | 0.4214 | 163.41 | 190.00 | 181.72 |  |
| **2** | 0.206 | 0.2598 | 0.2421 | 0.3018 | 0.5012 | 0.4755 | 146.50 | 192.92 | 196.41 |  |
| **3** | 0.2204 | 0.2187 | 0.2331 | 0.266 | 0.4562 | 0.412 | 120.69 | 208.60 | 176.75 |  |
| **4** | 0.2715 | 0.2577 | 0.2867 | 0.4359 | 0.638 | 0.6599 | 160.55 | 247.57 | 230.17 |  |
| **5** | 0.1872 | 0.2394 | 0.1805 | 0.2803 | 0.2945 | 0.4073 | 149.73 | 123.02 | 225.65 |  |
| **6** | 0.2028 | 0.2434 | 0.2047 | 0.3692 | 0.5515 | 0.5971 | 182.05 | 226.58 | 291.70 |  |
| **7** | 0.2563 | 0.2615 | 0.1938 | 0.4195 | 0.4676 | 0.5377 | 163.68 | 178.81 | 277.45 |  |
| **8** | 0.2778 | 0.2479 | 0.2763 | 0.3937 | 0.3719 | 0.4608 | 141.72 | 150.02 | 166.78 |  |
| **9** | 0.2153 | 0.2314 | 0.189 | 0.3347 | 0.4183 | 0.5312 | 155.46 | 180.77 | 281.06 |  |
| **10** | 0.2484 | 0.246 | 0.2142 | 0.383 | 0.387 | 0.3928 | 154.19 | 157.32 | 183.38 |  |
| **11** | 0.2422 | 0.2456 | 0.2186 | 0.3278 | 0.3501 | 0.448 | 135.34 | 142.55 | 204.94 |  |
| **12** | 0.2594 | 0.2539 | 0.3251 | 0.4413 | 0.5557 | 0.6114 | 170.12 | 218.87 | 188.07 |  |
| **13** | 0.2226 | 0.2745 | 0.2803 | 0.3768 | 0.4645 | 0.5334 | 169.27 | 169.22 | 190.30 |  |
| **14** | 0.2726 | 0.2508 | 0.236 | 0.4729 | 0.5288 | 0.5678 | 173.48 | 210.85 | 240.59 |  |
| **15** | 0.2883 | 0.2945 | 0.2225 | 0.3737 | 0.4314 | 0.5157 | 129.62 | 146.49 | 231.78 |  |
| **16** | 0.2829 | 0.2368 | 0.2042 | 0.4046 | 0.4359 | 0.4792 | 143.02 | 184.08 | 234.67 |  |
| **17** | 0.2835 | 0.273 | 0.2797 | 0.4489 | 0.4411 | 0.7034 | 158.34 | 161.58 | 251.48 |  |
| **18** | 0.2715 | 0.2013 | 0.2221 | 0.4104 | 0.3619 | 0.4519 | 151.16 | 179.78 | 203.47 |  |
| **Mean± SD** | 0.2485 ±0.0318 | 0.2495 ±0.021 | 0.2356 ±0.0393 | 0.3817 ±0.0594 | 0.4521 ±0.0843 | 0.5115 ±0.0896 | **153.80 ±15.92** | **181.61 ±32.45** | **219.80 ±38.00** |  |

**Table S1.** CT Measurements of Lumbosacral Foraminal Area Raw Data

^a^ Calculated as (Post-operative measurement / Pre-operative measurement) × 100
